# Transitional Needs of Australian Young Adults With Type 1 Diabetes: Mixed Methods Study

**DOI:** 10.2196/diabetes.8315

**Published:** 2017-11-30

**Authors:** Ashley H Ng, Timothy C Crowe, Kylie Ball, Bodil Rasmussen

**Affiliations:** 1 School of Nursing and Midwifery Deakin University Burwood Australia; 2 School of Exercise and Nutrition Science Deakin University Geelong Australia; 3 Institute for Physical Activity and Nutrition School of Exercise and Nutrition Science Deakin University Geelong Australia; 4 Centre for Quality and Patient Safety Research School of Nursing and Midwifery Deakin University Geelong Australia

**Keywords:** type 1 diabetes, mHealth, young adults, transition

## Abstract

**Background:**

Young adulthood is marked by transitions that impact diabetes self-management behaviors, which require ongoing diabetes education and support. Traditional diabetes education programs and services currently do not meet the needs of many young adults with type 1 diabetes mellitus (T1DM) as they continue to fall through the cracks of clinical services. Age-centered diabetes education programs and services present an opportunity for young adults to meet in a supportive environment and gain a better understanding about diabetes management.

**Objective:**

The aim of the study was to identify the health and well-being needs of Australian young adults aged between 18 and 35 years with T1DM to develop appropriate solutions to keep them engaged with diabetes self-management.

**Methods:**

In total, 13 semistructured individual interviews and self-reported surveys were obtained to understand participants’ experiences with diabetes education programs and services. Together with survey data, transcribed interviews were analyzed into themes and categories using comparative analysis to identify the health and well-being needs of young adults with T1DM during young adulthood.

**Results:**

Diabetes education and service needs for young adults with T1DM related to improving access to existing diabetes education programs and services, having credible informational resources, as well as having personalized diabetes management advice. Participants especially valued relevant and real-time information and opportunities for peer support, mostly sourced from Web-based platforms.

**Conclusions:**

There is a need for diabetes education programs and services to be age-appropriate and easily accessible, to provide relevant and credible information, and to provide opportunities for peer support to better support young adults with T1DM. These findings also support the use of diabetes education programs or services delivered online through mHealth systems in this population.

## Introduction

Young adults with type 1 diabetes mellitus (T1DM) have different needs compared with the pediatric and older adult populations as they are required to adapt their diabetes management against more dynamic and significant turning points encountered in young adulthood [1]. Turning points can be significant life events that represent a change in an individual’s life pattern, such as starting university, full-time employment, moving out of home, developing new relationships, or starting a family [1]. These critical life events are often stressful and can impact a young adult’s diabetes-related decision-making process [1].

### Navigating Through Life Transitions

As young adults navigate through life transitions, they are more likely to experience depressive symptoms and diabetes distress compared with older adults [2,3]. Additionally, competing life demands may ultimately displace self-care and diabetes management from being a priority, which may result in glycemic control deterioration [1,4]. Significant health-related turning points such as developing diabetes-related complications may further impact young adults’ diabetes management [1].

As young adults start to adapt and transition through turning points presented during the tumultuous period of young adulthood, they begin to acknowledge the importance and long-term health benefits that accompany an investment in self-care [4]. With renewed motivation, young adulthood presents as an opportunity for diabetes education programs and services to engage young adults who are ready for positive behavior change [4].

Diabetes education programs and services are crucial to promote health literacy in people with diabetes through the provision of skills and knowledge for efficient diabetes self-management [5]. Health literacy is a concept that expands beyond literacy and numeracy skills to how individuals access, understand, and use information to make appropriate decisions to promote and maintain good health [6,7]. Poor health literacy has been associated with inadequate health-related knowledge, lack of social support, and poor communication skills with health care professionals [8]. Collectively, these factors correlate with increased rates of hospitalization and use of emergency services [8]. Low levels of health literacy have also been associated with a lower motivation to engage in diabetes self-management and greater communication difficulties with health care professionals [9].

### Meeting the Needs of Australian Young Adults With Diabetes

Unfortunately, current diabetes education programs and services do not meet the needs of Australian young adults with T1DM [10]. Young adults face a host of barriers, which discourages access to current diabetes education programs and services [11]. Common barriers to access can be separated into logistical challenges, such as time constraints, cost, and distance needed to travel, and emotional barriers, such as feeling disempowered and disillusioned from appointments and a fear of judgment from health care professionals [11]. Enablers to diabetes education programs and services access corresponded with previously identified barriers such as continuity of care, time of day of clinical appointments, and proximity of clinic to home [11]. Additionally, although enablers to engage young adults in diabetes education programs and services have been identified and suggested to diabetes clinics, subsequent implementation status of these changes or patient outcomes is not known [11].

A major concern associated with low engagement in diabetes education programs and services is the lack of health care professional support for young adults with diabetes as they progress from pediatric health care services [10]. From the 2012 Australian Youth Transition Survey, it was found that only 42% of young adult respondents with diabetes aged between 18 and 24 years attended a diabetes clinic, which is the primary source of diabetes education provided to this population [10]. In contrast, 71% of adolescent respondents aged between 14 and 17 years attended a diabetes clinic [10]. A majority of young adults with diabetes who do not regularly receive clinical follow-up may subsequently have poorer glycemic control and increased risk of diabetes-related complications [11]. Low diabetes clinic attendance rates also reflect the lack of appropriate diabetes education program and services tailored for young adults [10].

Young adulthood is a complex period for young adults with T1DM where additional ongoing support would be of benefit as they learn to balance diabetes self-management with dynamic life changes. However, there is a paucity of research to inform diabetes education programs and service delivery to support and empower young adults with T1DM with their diabetes self-management as they transition through turning points. The aim of this study was to identify the diabetes education and service needs of young adults with T1DM during these turning points.

## Methods

### Study Design

A mixed-methods approach that included semistructured interviews and cross-sectional surveys was utilized to gain an understanding of the health and well-being needs of young adults with T1DM. Concepts derived from constructivist grounded theory influenced the qualitative approach of the study [12]. Cross-sectional surveys provided additional quantitative information around participants’ health literacy levels, emotional well-being, and diabetes distress level. Ethics approval was granted by Deakin University Human Research Ethics Committee, and the study has fulfilled the consolidated criteria for reporting qualitative research (COREQ) guidelines for reporting [13].

### Participants and Recruitment

Participants were Australian young adults living with T1DM aged between 18 and 35 years. Inclusion criteria included English proficiency and access to the Internet either through a smartphone or through a Web browser to complete a Web-based survey. There were no predetermined exclusion criteria. Recruitment was conducted online through flyers that were posted on relevant Australian diabetes-related social media channels and online support groups. Snowball sampling was used for recruitment until data saturation was achieved from qualitative interviews.

### Semistructured Interviews

Interviews were offered in person or online and took approximately an hour to complete. A set of guided questions included topics around events that impacted participants’ diabetes management and their experiences and recommendations to improve diabetes education programs and services. The question guide was reviewed and piloted by independent researchers to ensure questions were easy to understand and follow. Interviews were organized and conducted by AN, a PhD candidate with a dietetics background and lived experience with diabetes. AN’s background was only disclosed to participants when she felt it would help develop rapport. Face-to-face interviews were conducted at Deakin University (Burwood, Australia). A secure Web-based communication platform, Zoom videoconferencing software (California, United States of America), was used for Web-based interviews. To minimize bias from AN’s lived experience with diabetes, all interviews were audiotaped and field notes were recorded to critically reflect on the guided questions and interview to ensure objectivity was maintained. Guided questions were further refined if necessary after discussion of field notes with BR. After the interviews, participants were sent a one-page summary by email of the main points discussed during the interview to confirm and validate the data through a process called member-checking [14]. Participants were then asked to complete a Web-based survey, which was a compilation of validated questionnaires to gather information on their level of health literacy, emotional well-being, diabetes distress, and demographic details. The Web-based survey was administered through Qualtrics (Utah, United States of America), which uses a secure cloud storage platform.

### Health Literacy Questionnaire

The health literacy questionnaire (HLQ) is a self-administered 44-item questionnaire, which covers 9 domains of health literacy concepts that support effective self-management of chronic health conditions [15]. The 9 domains are as follows: (1) feeling understood and supported by health care providers, (2) having sufficient information to manage health, (3) active management of health, (4) social support for health, (5) appraisal of health information, (6) ability to actively engage with health care providers, (7) navigating the health care system, (8) ability to find good health information, and (9) understanding health information well enough to know what to do. Scores were averaged for each domain with scores toward the higher end of the scale indicating strength, or greater literacy, within that health literacy domain.

### Diabetes Distress Scale

The Diabetes Distress Scale (DDS) is a 17-item self-administered questionnaire, which comprises the following 4 subscales that contribute to overall diabetes distress: emotional distress, physician-related distress, regimen-related distress, and diabetes-related interpersonal distress [16]. DDS uses a 5-point Likert scale to rate statements related to living with diabetes. A higher overall score indicates greater diabetes-related distress, whereas subscale scores provide an indicator to the primary source of concern.

### World Health Organization-5 Well-Being Index

The World Health Organization-5 Well-Being Index (WHO-5) consists of 5 positively worded items relating to mood, vitality, and general interest over a 2 week period [17]. The self-administered questionnaire uses a 6-point Likert scale where participants are asked to rate the frequency of experiencing a feeling. Summated scores are converted into a percentage; with scores below 50 indicating a low mood and scores of 28 and lower indicating the likelihood of depression.

### Well-Being Questionnaire 12

The Well-being Questionnaire 12 (W-BQ12) is a 12-item self-administered questionnaire, which uses a 4-point Likert scale to describe how often participants experienced a feeling in the past few weeks [18]. For this study, the 4-item positive well-being subscale of the W-BQ12 was used. A higher overall score correlates with greater general well-being.

### Data Analysis

Interviews were recorded and transcribed by AN and analyzed using NVivo (QSR International, Australia, Melbourne). Transcripts from interviews underwent line-by-line coding, categorization, and subcategorization in line with the process of constructivist grounded theory as described by Charmaz [19]. Codes with similar meaning or context identified through line-by-line coding from the interviews were integrated into a category or theme by AN in a process known as thematic comparative analysis. Through this process, turning points experienced by participants as well as their associated transitional needs to be able to successfully transition through those turning points were identified. Categories and subthemes were reviewed by BR and TC, and discrepancies were discussed until a consensus was reached. A one-page summary of findings from the interviews was then provided to participants for further feedback.

Descriptive statistics were used to analyze demographic and other quantitative cross-sectional data from the Web-based questionnaire.

## Results

### Participant Characteristics

From 20 initial expressions of interest, 13 participants completed both the interview and the Web-based survey. Reasons cited for nonparticipation included unwilling to complete an interview and lack of time. On average, participants were predominantly female, aged between 19 and 35 years, lived with diabetes between 2 and 25 years, and managed their diabetes with an insulin pump. Other participant characteristics are described in Table 1.

**Table 1:** Summary of participant characteristics (N=13).

Characteristics		Participants with T1DM^a^
**Gender**		
	Female, n (%)	10 (77)
	Male, n (%)	3 (23)
Age in years, mean (SD^b^)		20 (5)
Diabetes duration in years, mean (SD)		8 (6)
**Diabetes management**		
	Insulin pump, n (%)	8 (62)
	Multiple daily injections, n (%)	5 (38)
**Residential area**		
	Metropolitan area, n (%)	10 (77)
	Regional area, n (%)	2 (15)
	Undisclosed, n (%)	1 (8)
**Employment status**		
	Working full time, n (%)	7 (53)
	Studying full time, n (%)	3 (23)
	Studying part time, n (%)	1 (8)
	Other, n (%)	1 (8)
	Undisclosed, n (%)	1 (8)

^a^T1DM: type 1 diabetes mellitus.

^b^SD: standard deviation.

### Health Literacy and Emotional Well-Being

Table 2 summarizes participant scores across all quantitative measures within the Web-based survey, which includes the HLQ, DDS, W-BQ12, and WHO-5. On average, participants scored toward the higher end across each HLQ domain, indicating adequate health literacy levels toward self-management. Average scores from the DDS and subscales were not indicative of any overall moderate diabetes distress. However, individual scores demonstrated that moderate distress existed within each domain, with moderate distress reported by 75% (9/12) of participants within the emotional burden subscale, 50% (6/12) of participants within the interpersonal burden subscale, 33% (4/12) of participants within the regimen burden subscale, and 25% (3/12) of participants within the physician domain subscale. Overall, participants reported satisfactory emotional well-being and positive well-being.

Key themes identified from the qualitative data fell into two major categories and included events considered as turning points in participants’ diabetes management and transitional needs to successfully navigate a turning point. The following section will focus on the transitional needs of young adults and subthemes identified, supported with descriptive quotes.

### Transitional Needs

As participants described their transition through turning points, they highlighted several factors or needs that enabled them to modify their diabetes self-management to cope and adapt. These transitional needs included receiving support from health care professionals; awareness of and easier access to existing diabetes education programs and services; access to credible, relevant, and timely information; and peer support.

#### Health Care Professional Support

Although 8 participants acknowledged the value of health care professionals in their diabetes management, they described an absence of rapport due to a lack of continuity of care. As a result, participants expressed difficulty in the ability to communicate their needs with health care professionals. Participants who are within the public health care system had no guarantee to see the same health care professional during clinical appointments. Without rapport, young adults reported being less likely to receive personalized health advice in response to their current needs and circumstances from health care professionals. One participant stated:

There’s no ongoing relationship. It’s like seeing a new GP fresh from the start every time you go in. So, we see a new specialist and you’d have to keep giving the background every time and it doesn’t get to the point where they get to know you well enough to understand what could help you. So, I find that it is a bit of a waste of time.

Female, 29

**Table 2:** Participant scores across quantitative measures.

Quantitative measures		Mean (SD^a^)
**Health literacy questionnaire domains (n=13)**		
	1. Feeling understood and supported by health care professionals	3.06 (0.58)
	2. Having sufficient information to manage my health	3.02 (0.55)
	3. Actively managing my health	3.31 (0.56)
	4. Social support for health	2.80 (0.61)
	5. Appraisal of health information	2.91 (0.36)
	6. Ability to actively engage with health care providers	3.38 (0.53)
	7. Navigating the health care system	3.37 (0.53)
	8. Ability to find good health information	3.63 (0.71)
	9. Understanding health information well enough to know what to do	4.02 (0.49)
**Diabetes distress scale (n=12)**		
	Emotional burden	2.83 (1.03)
	Physician burden	1.93 (0.71)
	Regimen burden	2.08 (0.70)
	Interpersonal burden	2.42 (1.04)
	Total score	2.32 (0.72)
**Emotional well-being (n=12)**		
	Well-being questionnaire 12 4-item subscale	59.33 (19.88)
	World Health Organization-5 Well-Being Index	6.33 (3.31)

^a^SD: standard deviation.

Out of the participants, 4 young adults reported feeling judged by health care professionals during clinical appointments when their lab results were outside target ranges. Participants perceived that health care professionals assumed young adults with poor glycemic control held negative attitudes toward their health. One participant stated:

I have had negative comments and feedback from [health care professionals] who haven’t bothered to understand why I haven’t controlled my diabetes. They’ve kind of just made judgements and made me feel like I’m failing with my diabetes.

Female, 30

#### Awareness of Diabetes Education Programs and Services

Of the participants, 7 reported that they were not always aware of available diabetes education programs, or services offered to them, which could inform their diabetes management choices. Additionally, 3 participants described that most topics offered through diabetes-related information sessions or workshops were not relevant to their current needs or situation. Consequently, participants were less likely to attend diabetes services if they perceived them to be of little value. One participant stated:

I know there is a little bit of a dead spot at this sort of age group. You get a whole lot of support when you’re an adolescent and a child and for things like pregnancy. But I think you definitely have to go looking if you’re a young person with type 1 diabetes.

Female, 24

Participants were more likely to hear of a diabetes education program or service through their local diabetes organization or social networks rather than during appointments. However, one participant still felt that diabetes information sessions or peer support were not readily promoted to those who would benefit from them. Another participant believed that it would be better received by young adults and more appropriate for health care professionals to connect them with upcoming diabetes information sessions and services. One participant stated:

I don’t think [diabetes education programs and services] as widely advocated by the health care professionals. But I think there’s a wide range if you know what you’re looking for and you need to know someone who’s kind of promoting these things.

Female, 35

Unsurprisingly, 5 participants agreed that it was crucial to improve awareness around existing diabetes education programs and services to young adults with diabetes. Although participants understood the importance of such services in helping people to cope or adapt to life with diabetes after accessing them, they felt that it needed further emphasis to reach those who need it most. One participant stated:

The biggest suggestion I would have is letting people know that [diabetes education programs and services are] available. Through social networking, I guess, promoting its importance and doing that not only through social networks but through GPs, through endocrinologists, through other people with diabetes out there.

Male, 35

Although participants highlighted the desire for health care professionals to promote greater awareness of diabetes services, they valued their independence to seek out such services at a time when they feel ready. Turning points are a time where young adults report feeling motivated and ready to engage in health behavior change and therefore present as a prime opportunity to promote diabetes education programs and services. Of the participants, one shared her experience of completing the Dose Adjustment for Normal Eating (DAFNE) workshop for the second time as an example of the importance of timing to be engaged with information presented for effective education:

To do education like DAFNE you need to be in the right mindset, you need to be engaged and have a little bit of motivation to take the information on board. Or you can go and do the course and come away thinking I wasn’t in the right space to do it, which was why I did it for the second time.

Female, 30

#### Diabetes Education Program and Service Access

Participants reported that they were aware of some diabetes education programs and services they could access. However, a significant challenge encountered by 6 participants was the ability to find time to attend clinical appointments or diabetes services, such as peer support groups or educational sessions. Participants talked about the need to juggle multiple commitments against a busy lifestyle, which made it difficult for them to prioritize a continued investment into their health. Services offered by the public health care system often fell within business hours, alongside participants’ other commitments such as work and study. Participants perceived taking leave from paid work or study time as a major disruption to their career development. One participant stated:

I work full time obviously, like most people, so it’s hard to get away and get the time to seek out [diabetes education] as well.

Male, 35

When participants did access clinical services, they expressed frustration at long wait times to see health care professionals. Especially within the public health care system, participants reported spending up to 3 hours in the waiting room before being attended to. Although diabetes services offered beyond clinical settings were reported to be readily available, they only benefited those who lived within metropolitan areas. Due to the long travel distance, young adults who lived in regional areas missed out on valuable diabetes-related information events, which could help them navigate turning points such as addressing their mental health. One participant stated:

I get some things in the mail occasionally for seminars…but I had moved 2 hours away and so it was more of an inconvenience to go. So, I never really went.

Female, 21

Another participant stated:

I think mental health is important and the stress of diabetes can affect some people…I was never offered any mental health support or help that I needed. It was never there.

Male, 33

As an alternative to face-to-face services, 8 participants discussed using the Internet to search for diabetes-related information when they were unable to access relevant information sessions or ask their health care professional.

One participant stated:

It’s very helpful to be able to look up a website and get the information that you need.

Female, 34

Another participant stated:

I probably get information online more than anything else.

Female, 24

#### Access to Credible, Relevant, and Timely Information

Turning points are dynamic events in young adults’ lives during which participants have reported a need for real-time and ongoing support outside clinical appointments. In total, 9 participants stated that they turned to alternative sources for diabetes-related information, which largely included online search engines and social media, when health care professionals were unavailable. For these young adults, the convenience of having information at their fingertips through their smartphones was well suited to meet their needs when transitioning through turning points. Additionally, participants who are on the cusp of a transition were able to search for relevant information discreetly in their own time. Participants also regarded Web-based sources of information and flexible communication methods as being cost- and time-effective compared with physically attending an appointment with their health care professional.

One participant stated:

I wish I had an educator that I could drop a quick email or a text or a phone call to ask, “can you help me with this.” I definitely wouldn’t have needed a face-to-face appointment but having that access to somebody in a professional capacity would be good.

Female, 30

Another participant stated:

If I had to find something out that I couldn’t get an answer from the public hospital system, I would Google it. I’ve had to Google to find out what my sugar levels would do at altitudes because I was going overseas.

Female, 29

Despite the heavy reliance on Web-based sources for diabetes-related information, participants questioned the reliability and credibility of the information they would come across. Of the participants, 5 stated that they felt competent enough to filter inaccurate information through their own experience and common sense.

One participant stated:

The information on the net is really unreliable. I mean some of it is great but you don’t know what’s accurate and what’s not. What’s kind of a peer-reviewed article versus somebody stating this happened to me therefore it is the case for all people with diabetes. There’s reliable and then there’s blogs, which can be helpful in hearing what other people have experienced.

Female, 32

On the contrary, some participants felt intimidated by the wealth of information on the Internet, which further compounds on the often-stressful nature of transitioning through a turning point. Subsequently, participants lacked the confidence to identify evidence-based facts and credible Web-based sources as highlighted by a young adult:

You do a search on Google and you don’t even know where to begin. Like what’s real and what’s reputable…so I wouldn’t know where to begin.

Female, 32

Participants valued evidence-based updates around various aspects of diabetes management, such as nutrition and exercise, as they transition toward positive health behavior changes in response to a maturation in their perception of health. Specifically, 6 participants wanted practical tips around making healthy food choices, managing diabetes around alcohol, and information on how different exercises affect blood glucose levels.

One participant stated:

The right [nutrition] messages aren’t that easy to interpret because people go to the shops and something claims to be healthy but it’s not necessarily healthy.

Male, 35

Additionally, as participants explored various diabetes management strategy options to adapt to changes in their lives, 4 young adults were keen to receive more information and user reviews of various diabetes technologies available in Australia. However, some participants found it challenging to navigate through the plethora of information available online. One participant stated:

There is so much [information online]. It’s hard to sift through what’s going to be useful and what’s not and there’s a lot of people making money off it as well. So, it would be nice to know what programs and what meters are useful, all of that sort of thing.

Female, 24

Participants also expressed the need for tips and guidance around factors that indirectly affect diabetes management such as budget assistance and mental health support. Among the participants, 4 stated that health care professionals generally failed to recognize the importance of emotional well-being in diabetes management. Consequently, participants felt isolated as they attempted to navigate information or services to help them cope or validate the emotional aspect that accompanies living with chronic condition. One participant stated:

I think having psychological support isn’t something I’ve found generally offered but it’s something that everybody needs regardless of what age you’ve been diagnosed.

Female, 30

Overall, majority of participants wanted easy access to credible diabetes-related information, through an easy-to-navigate Web-based resource. Convenience and perceived value were important considerations to participants, especially during stressful periods as they transition through a turning point. Young adults needed to be reassured that their efforts in their search for information would not be wasted. One participant stated:

[I would like] something that I can access at 5 am in the morning when I’m feeding my baby and have got nothing else to read. It sounds really bad but things that can fit into my lifestyle because I don’t have time.

Female, 34

#### Peer Support

Overall, 7 participants regarded peer support as a highly valuable source of information and support in general as well as through turning points. Primarily, participants appreciated the ability to relate with others who understood or have experienced similar turning points, which reduces the sense of isolation. As a result, participants credited peer support as a positive impact on their emotional well-being through reciprocal support and encouragement toward their own diabetes management. Such a level of understanding and support was rarely received from health care professionals, unless the participant’s health care professional, often a diabetes educator, also lived with diabetes.

One participant stated:

It’s kind of nice knowing people who understand if you’re having a tough time or you need a little bit of advice, knowing someone else who lives with diabetes is probably more likely to understand than a health care professional.

Female, 30

Another participant stated:

I spoke to a few people [with diabetes] and said I drank too much one night and they said “yeah, well I’ve done that heaps of times.” It felt good to go “oh, I’m not alone in this.”

Male, 33

## Discussion

This study aimed to identify the health and well-being needs of young adults with T1DM during life transitions. Although it appeared that quantitative measures of health literacy, emotional well-being, and diabetes distress, on average, did not reflect major difficulties faced by participants in their diabetes management, individual scores showed otherwise. These findings were further supported by the qualitative data. Additionally, a significant gap in appropriate and relevant diabetes education and services for this population was highlighted through qualitative interviews. Transitional needs to manage turning points identified by participants included health care professional support, improved awareness and access to current diabetes services, resources with credible and relevant information delivered in a timely manner, and opportunities for peer support.

### Principal Findings

Turning points are often described as stressful events in a young adult’s life, which can result in glycemic changes that subsequently affect their well-being [1]. For a positive transition from a turning point to occur, the needs of young adults must be addressed through breaking down barriers for them to access the relevant information and support they require [20].

Young adults are required to balance competing life demands while they navigate turning points, which may displace self-care behaviors from being a priority [1,21]. Erratic schedules are commonly seen within the young adult population, which further compounds on positive health behavior required for diabetes self-management [22,23]. As majority of diabetes education programs and services are provided during typical business hours, participants considered attendance to be intrusive on their schedules, especially if the value of attendance is perceived to be low. Findings from this study suggest that the use of Web-based events held outside business hours and flexible communication methods with health care professionals may overcome these logistical barriers young adults face in attending diabetes education programs and services.

Apart from logistical barriers, many identified transitional needs that are closely aligned with the definition of health literacy and the HLQ [15]. For example, participants scored the lowest within the *social support for health* domain of the HLQ, which was reflected as they emphasized the importance of peer support to aid transitions through turning points. Psychosocial support motivates individuals toward positive health behavior changes and has been continuously recognized for its positive impact on emotional well-being [24,25]. Peers with diabetes understand and relate to the unique challenges of living with diabetes through their own experiences [1]. As they share past experiences, peers provide practical informational support, which helps other young adults with diabetes to build upon their problem-solving skills [24]. The importance of peer support was highlighted in this study as young adults described how connecting with other people with diabetes removed the isolation of living with T1DM through their shared experiences. Through the creation of a sense of normality for young adults with T1DM, they are further encouraged to continue with their efforts of diabetes self-management.

Effective communication between health care professionals and people with diabetes preface the concept of patient-centered care, which is closely intertwined with health literacy [26]. Patient-centered care posits that the person living with diabetes should lead the way in his or her self-management plan, under the guidance and support of health care professionals [26]. Participants often encountered a lack of continuity of care within the health care system and found it a challenge to build rapport with health care professionals. As a result, young adults perceive that without rapport, health care professionals are less likely to provide personalized diabetes management advice, which is relevant to their changing needs and circumstances. These findings are concerning as previous research identified that young adults who were made to feel disempowered or disillusioned by their health care professionals are then less likely to attend a follow-up appointment [11].

Life transitions during young adulthood comprise several sensitive topics such as experimenting with alcohol, managing diabetic ketoacidosis episodes, and starting a family, including contraceptive options [27]. Without readily available access to relevant diabetes education and services, a majority of participants turned to Web-based sources for diabetes-related information. The Internet allows young adults to maintain anonymity as they search for information required to make decisions in relation to their turning points [27]. Web-based sources of health-related information hold several advantages such as the absence of physical and geographical limitations to access, a sense of anonymity, and its cost-effectiveness. [1,28]. However, some participants reported lack of confidence to identify credible sources of information, which relates to the *appraisal of health information* domain of the HLQ, the second lowest scoring domain in this study.

### Strengths and Limitations

Although inferential statistics could not be drawn from quantitative measures because of the small sample size, its data provided additional value to the qualitative data drawn from participants’ interviews. As such, the main strength of the study was the use of a mixed-methods approach to determine the health and well-being needs of young adults with T1DM.

### Conclusions

Young adults living with T1DM would benefit from learning to adapt their diabetes management to cope with a host of significant life- or health-related events. As young adults encounter a turning point that subsequently impacts their health, there can be an increase in motivation for positive health behavior change. However, adequate support from health care professionals and peers; access to appropriate, credible, and timely information resources; and targeted diabetes education and services tailored for young adults are required to enable a successful transition from a turning point. Findings from this study highlight a significant gap within present diabetes education programs and services and put forward the benefits in the use of mHealth for young adults with T1DM during life transitions.

## Abbreviations

DAFNE: Dose Adjustment for Normal Eating
DDS: Diabetes Distress Scale
HLQ: Health Literacy Questionnaire
T1DM: type 1 diabetes mellitus
W-BQ12: Well-being Questionnaire 12
WHO-5: World Health Organization-5 Well-Being Index
